# Matrine displayed antiviral activity in porcine alveolar macrophages co-infected by porcine reproductive and respiratory syndrome virus and porcine circovirus type 2

**DOI:** 10.1038/srep24401

**Published:** 2016-04-15

**Authors:** Na Sun, Panpan Sun, Haipeng Lv, Yaogui Sun, Jianhua Guo, Zhirui Wang, Tiantian Luo, Shaoyu Wang, Hongquan Li

**Affiliations:** 1 College of Animal Science and Veterinary Medicine, Shanxi Agricultural University, Taigu 030801 Shanxi, P. R. China; 2 Department of Pathobiology, College of Veterinary Medicine, Texas A&M University, College Station, Texas, USA; 3 Center for Transplantation Sciences, Massachusetts General Hospital and Harvard Medical School, Boston MA, USA; 4 Shanxi Veterinary Prevention and Treatment Station, Taiyuan 030024, P. R. China; 5 School of Community Health, Faculty of Science, Charles Sturt University, Orange NSW, Australia

## Abstract

The co-infection of porcine reproductive respiratory syndrome virus (PRRSV) and porcine circovirus type 2 (PCV2) is quite common in clinical settings and no effective treatment to the co-infection is available. In this study, we established the porcine alveolar macrophages (PAM) cells model co-infected with PRRSV/PCV2 with modification *in vitro*, and investigated the antiviral activity of Matrine on this cell model and further evaluated the effect of Matrine on virus-induced TLR3,4/NF-κB/TNF-α pathway. The results demonstrated PAM cells inoculated with PRRSV followed by PCV2 2 h later enhanced PRRSV and PCV2 replications. Matrine treatment suppressed both PRRSV and PCV2 infection at 12 h post infection. Furthermore, PRRSV/PCV2 co- infection induced IκBα degradation and phosphorylation as well as the translocation of NF-κB from the cytoplasm to the nucleus indicating that PRRSV/PCV2 co-infection induced NF-κB activation. Matrine treatment significantly down-regulated the expression of TLR3, TLR4 and TNF-α although it, to some extent, suppressed p-IκBα expression, suggesting that TLR3,4/NF-κB/TNF-α pathway play an important role of Matrine in combating PRRSV/PCV2 co-infection. It is concluded that Matrine possesses activity against PRRSV/PCV2 co-infection *in vitro* and suppression of the TLR3,4/NF-κB/TNF-α pathway as an important underlying molecular mechanism. These findings warrant Matrine to be further explored for its antiviral activity in clinical settings.

Porcine circovirus associated diseases (PCVAD), causing large economic losses for the swine industry in the world, is characterized by postweaning multi-systemic wasting syndrome (PMWS), porcine respiratory disease complex (PRDC), porcine dermatitis and nephropathy syndrome (PDNS), proliferative and necrotizing pneumonia (PNP), enteric signs including diarrhea and weight loss, reproductive disorders including abortions, stillbirths and fetal mummification, and acute pulmonary edema (APE)^1^,^2^. PCVAD is a multifactorial disease linked to both non-infectious factors and infectious factors. The non-infectious factors including environmental conditions and management strategies that could be improved to reduce the disease occurrence. The infectious factors contained a variety of viral and bacterial pathogens such as porcine reproductive respiratory syndrome virus (PRRSV), porcine circovirus type 2 (PCV2), porcine parvovirus (PPV), swine influenza virus (SIV), or *mycoplasma hyopneumoniae*^3^,^4^,^5^. PRRSV is an enveloped, single stranded positive-sense RNA virus, which belongs to the family *Arteribiridae* of the order *Nidovirales*. PRRSV was first isolated in the United States in 1987 and Europe in 1990 which represents two major genotypes of PRRSV. In 2006, the highly pathogenic PRRSV (HP PRRSV) strains were reported in China, posing a significant threat to the global swine industry^6^,^7^. Porcine circovirus 2 (PCV2) is the smallest known animal viruses, belong to the genus *Circovirus* in the *Circoviridae* family. The first emergence of PCV2 was isolated from the diseased pigs associated with PMWS^8^. PRRSV and PCV2 are the most common pathogens of PCVAD and co-infection of PRRSV and PCV2 are quite common in clinical cases^5^.

Vaccination is traditionally considered as the most effective method for preventing viral diseases. However, the protection period of vaccine against disease is limited and the virus cannot be eradicated by vaccination^9^. Furthermore, no effective vaccines are available for preventing multifactorial disease such as PCVAD. So it is urgent to find alternative effective measures to control the disease.

Matrine (C_15_H_24_N_2_O) is an alkaloid extracted from *sophora flavescens Ait*., has been demonstrated to possess a wide range of pharmacological effects including antioxidant^10^, anti-tumor^11^, anticancer^12^, antiinflammatory^13^, and antinociceptive^14^. The chemical structure was shown in Fig. 1. A recent study showed that Matrine-like compounds exhibited antiviral activity against the Coxsackie virus B3 (CVB3) and influenza virus A/Hanfang/359/95 (H3N2)^15^, but mechanisms of such antiviral activity is not investigated. In our previous research, we have shown that Matrine inhibited PRRSV replication in Marc-145 cells^16^ and interfered with PCV2 infection in PK-15 cells^17^.

In present study, we aim to establish PRRSV/PCV2 co-infected PAM model, and then evaluate the antiviral activities of Matrine on PRRSV/PCV2 using this model.

## Results

### Cytotoxicity of Matrine and Ribavirin on PAM cells

**Figure 2:**
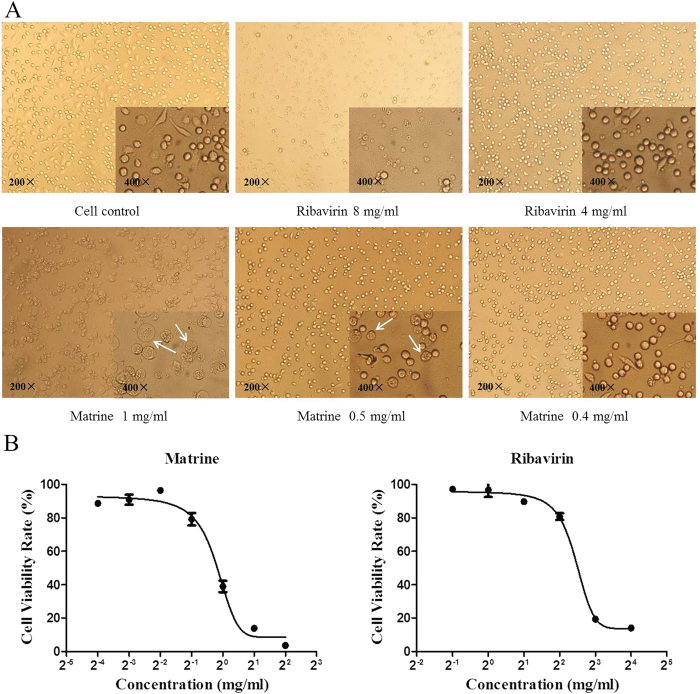
Cytotoxicity of Matrine and Ribavirin on PAM cells. PAM cells were incubated with different concentrations of Matrine and Ribavirin for 48 h. (**a**) the cell morphological changes by compound treatment. Typical vacuolization indicated by white arrows. (**b**) the cell viability rate was displayed “S” shape curve after treatment with Matrine or Ribavirin at different concentration, indicating the cytotoxicity of Matrine and Ribavirin on PAM cells was in a dose-dependent manner within the concentrations employed.

MTT method and the cytopathologic effect (CPE) monitored using microscopy were used to determine the maximum non-cytotoxic concentration (MNTC) and CC_50_ of Matrine and Ribavirin in PAM cells treated with different concentrations of Matrine and Ribavirin for 48 h. As shown in Fig. 2a, the cytotoxicity to PAM cells was closely related with the concentrations of Matrine and Ribavirin. Specifically, compared with cell only control, 1 mg/ml of Matrine treated PAM cells displayed remarkable morphological changes such as detachment of monolayer, cell disruption, particularly vacuolization (Fig. 2a, indicated by white arrows). Most of cells treated with 0.5 mg/ml were in good condition, but still had some cells showed vacuolization. Morphology of PAM cells treated with 0.4 mg/ml Matrine were the same as in cell control. the OD value of PAM cells treated with 0.4 mg/ml of Matrine from the MTT assay had no statistical differences with that of cell control. Hence, 0.4 mg/ml of Matrine was used as the safe concentration to cells in this studies. In contract to Matrine, treatment of PAM cells with 8 mg/ml of Ribavirin showed remarkable morphological changes such as detachment of monolayer, cell disruption and darkening of cell boundaries. No vacuolization was observed at Ribavirin treated cells, indicating that morphological difference between these two compounds. In addition, the cytotoxicity of Matrine and Ribavirin was found to be in a dose-dependent manner with the concentrations employed (Fig. 2b). Increasing concentrations of compounds will decrease the cell viability. The maximum non-cytotoxic concentration (MNTC) and CC_50_ values of Matrine and Ribavirin were listed in Table 1.

### Kinetics of PRRSV N gene and PCV2 CAP gene in PAM cells

**Figure 3:**
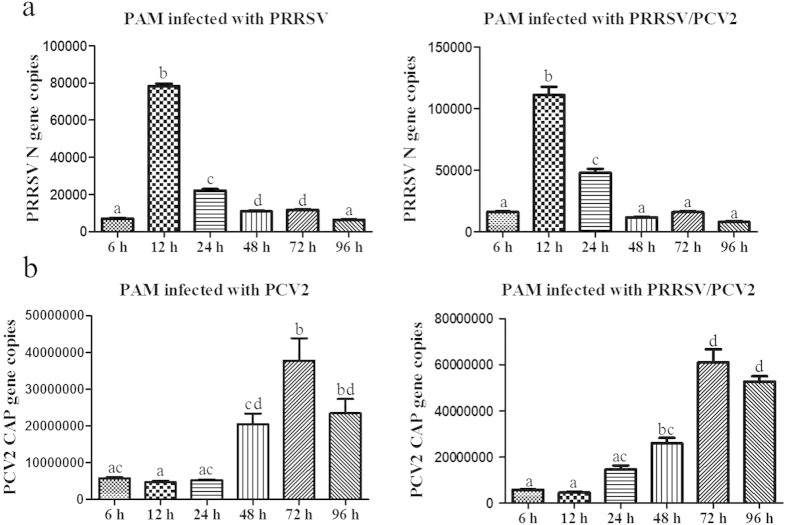
The detection of PRRSV N gene copies (**a**) and PCV2 CAP gene copies (**b**) respectively in PRRSV infected PAM cells, PCV2 infected PAM cells and PRRSV/PCV2 co-infected cells from 6 hpi to 96 hpi. The specific gene copies of PRRSV and PCV2 were determined by absolute quantitative real time PCR. Data were expressed as mean ± standard errors mean (SEM). Data with different letters (**a**–**d**) indicated that the group was significant different from other groups (*p* < 0.05).

**Figure 4:**
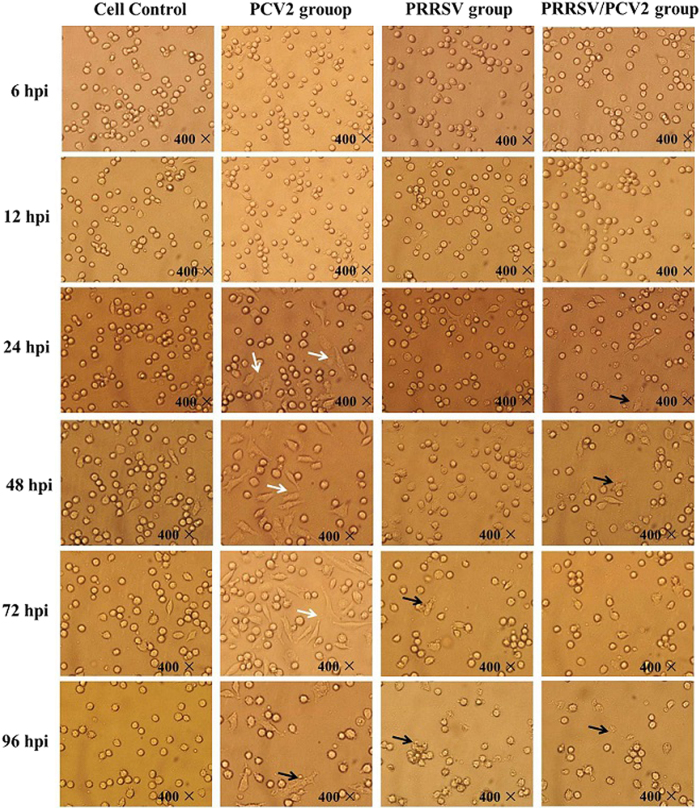
PAM cells respectively infected with PRRSV, PCV2 and PRRSV/PCV2 from 6 hpi to 96 hpi. Normal PAM cells displayed globular and clear cell boundary. At 96 hpi, in PRRSV alone group and PRRSV/PCV2 co-infection group, PAM cells presented various degree of morphological changes such as cell disruption, detachment of monolayer and aggregation (black arrows in Fig. 4). From 24 hpi to 72 hpi, the spindle cell formed in PCV2 alone infected PAM cells (white arrows in Fig. 4). Magnification:400×.

Viral copies were quantified from 6 hpi to 96 hpi to determine the kinetics of PRRSV and PCV2 replication in PAM cells. The variation trend of PRRSV N gene copies or PCV2 CAP gene copies in both PRRSV alone infected group or PCV2 alone infected group was basically consistent with that in PRRSV/PCV2 co-infected group from 6 to 96 hpi. As shown in Fig. 3a, the PRRSV N gene copies increased from 6 hpi to 12 hpi, and the N gene copies reached a peak at 12 hpi. Moreover, the N gene copies were gradually declined from 12 hpi to 96 hpi. Nevertheless, compared with 6 hpi, the N gene copy numbers were significant higher at 24 hpi (p < 0.05) both in PRRSV alone infected group and PRRSV/PCV2 co-infected group. Moreover, there were almost no remarkable morphological changes at 12 hpi, and the PAM cells displayed globular and intact cell boundary. However cell disruption and aggregation were observed from 24 hpi to 96 hpi of PRRSV infection alone or PRRSV/PCV2 co-infection (Fig. 4, pointed by the black arrows).

**Figure 5:**
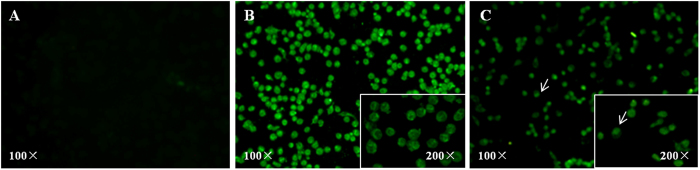
Detection of PCV2 CAP protein by IFA in PCV2 infected PAM cells. After 12 and 72 hpi, cells were fixed and then treated with 0.1% triton-100 in PBS for 15 min. After that the cells were blocked by 3% BSA, then incubated with rabbit anti-CAP monoclonal antibody at 37 °C for 2 h. After washing, cells were stained with Alexa Fluor 488 conjugated affinipure goat anti-Rabbit IgG for 1 h. The cells were examined by a fluoresence microsope. No specific immunofluoresence in Normal cells (**A**). PCV2 CAP protein was detected in the cytoplasm of the infected PAM cells at 12 hpi (**B**). At 72 hpi, the cap protein was detected in the nucleus of the infected PAM cells (**C**).

As shown in Fig. 3b, the PCV2 CAP gene copies were gradually increased with the longer incubation time of PAM cells infected with PCV2 or PRRSV/PCV2 (from 6 hpi to 96 hpi). Especially at 72 and 96 hpi, the CAP gene copy numbers were significant higher than that at other time points (p < 0.05). Moreover, the capsid protein was detected in the cytoplasm of the infected PAM cells at 12 hpi (Fig. 5b), and relocated to the nucleus by 72 hpi but detected in the lower number of infected cells (Fig. 5c). In addition, from 24 hpi to 72 hpi, the PAM cells infected with PCV2 alone were mainly fusiform instead of spherical cells (Typical figures were shown in white arrows in Fig. 4). At 96 hpi, PCV2 alone infected PAM cells showed cell disruption and detachment. However, the PRRSV/PCV2 co-infected PAM cells displayed mostly spherical during the entire incubation period from 6 hpi to 96 hpi.

### PRRSV/PCV2 co-infection enhanced PRRSV and PCV2 replication in PAM cells

**Figure 6:**
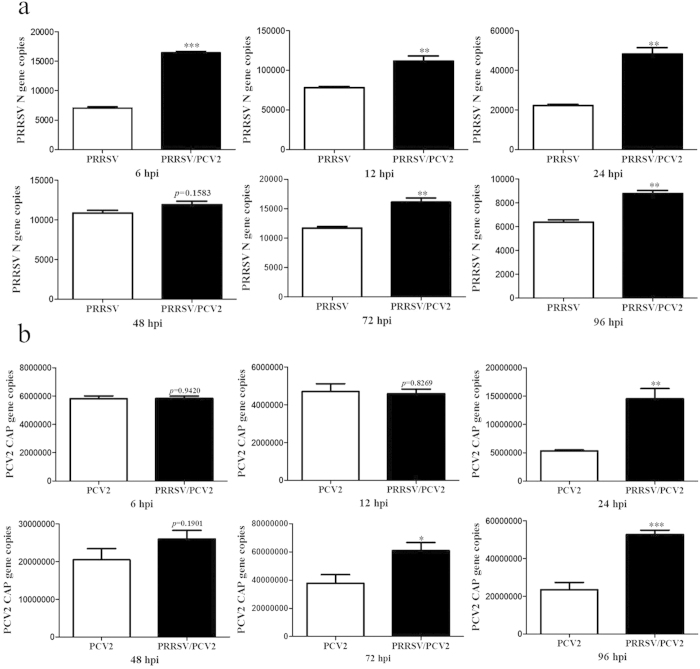
Detection PRRSV N gene copies in PRRSV infected PAM cells and PRRSV/PCV2 co-infected cells from 6 hpi to 96 hpi (**a**). Detection of PCV2 CAP gene copies in PCV2 infected PAM cells and PRRSV/PCV2 co-infected cells from 6 hpi to 96 hpi (**b**). The specific gene copies of PRRSV and PCV2 were determined by absolute quantitative real time PCR. Data were expressed as mean ± standard errors mean (SEM). *means p < 0.05, **indicates p < 0.01, ***means p < 0.001.

The PRRSV N gene copy numbers were much higher in PRRSV/PCV2 co-infected group than that in PRRSV alone infected group from 6 hpi to 96 hpi (p < 0.05). At 48 hpi, the N gene copies in PRRSV/PCV2 co-infected group were higher than that in PRRSV alone infected group, but not significantly (Fig. 6a). The PCV2 gene copies were significantly higher in PRRSV/PCV2 co-infected group at 24, 72 and 96 hpi (p < 0.05), compared with PCV2 alone infected group (Fig. 6b). There was no significant difference in genes copy number of PCV2 at 6, 12 and 48 hpi. Nevertheless, these results indicated that PRRSV/PCV2 co-infected cell model enhanced both PRRSV and PCV2 replication in PAM cells.

### Matrine inhibited PRRSV and PCV2 replication in PRRSV/PCV2 co-infected PAM cells

**Figure 7:**
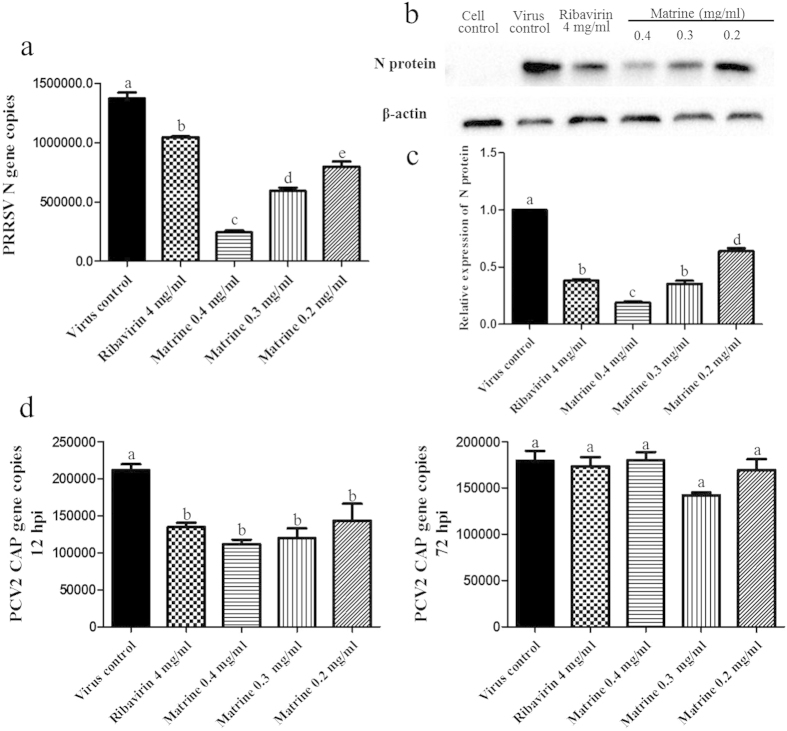
Inhibition of PRRSV and PCV2 gene expression in PRRSV/PCV2 co-infection by Matrine. Detection of PRRSV N gene (**a**) and protein (b and c) and of PCV2 CAP gene (**d**). Data were expressed as mean ± standard errors mean (SEM). Data with different letters (**a**,**b**,**c**,**d**,**e**) indicated that the group was significant different from other groups (*p* < 0.05).

In PAM cells, the PRRSV N gene copies reached the peak at 12 hpi, and the PCV2 CAP gene reached at 72 hpi. Therefore we performed the experiments both at 12 hpi and 72 hpi to assess the antiviral activity of Matrine. At 12 hpi, PAM cells from all treatment groups were collected to determine the expression of PRRSV N gene/protein using absolute quantitative real time PCR and Western blot analysis. The results were shown as Fig. 7. Compared with virus control, the N gene copies were significantly decreased in Matrine or Ribavirin treated group, especially in 0.4 mg/ml Matrine treated group (Fig. 7a). With the decreasing of Matrine concentrations, the N gene copies were increasing, but still lower than that in virus control, indicating that Matrine inhibited PRRSV N gene replication with a dose-dependent manner. The N gene copies in 4 mg/ml Ribavirin treated group was lower than that of virus control, but higher than that of Matrine treated groups, suggesting that Matrine possessed stronger anti-PRRSV activity than Ribavirin did. These results were confirmed by Western blot analysis (Fig. 7b,c). The expression of N protein in compounds treatment groups were much lower than that in virus control, and the level of N protein expression in 0.4 mg/ml Matrine treatment group was the least. Therefore, all these results provided clear evidences that Matrine inhibited PRRSV N gene/protein expression in PRRSV/PCV2 infected PAM cells model.

At 12 and 72 hpi, PCV2 CAP gene copy number in PAM cells from all treatment groups were determined using absolute quantitative real time PCR analysis. As shown in Fig. 7d, the CAP gene copies were significantly decreased in compound treatment groups at 12 hpi in comparison with virus control. There was no significant difference in inhibiting the CAP replication between Ribavirin treatment and Matrine treatment groups. At 72 hpi, there were no marked change in the PCV2 CAP gene copies between virus control and compound treatment groups. Taken all these results together, Matrine disturbed the expression of PRRSV N gene and PCV2 CAP gene in PRRSV/PCV2 infected PAM cells at 12 hpi.

### Matrine restrained virus induced TLR 3 and TLR 4 expression

**Figure 8:**
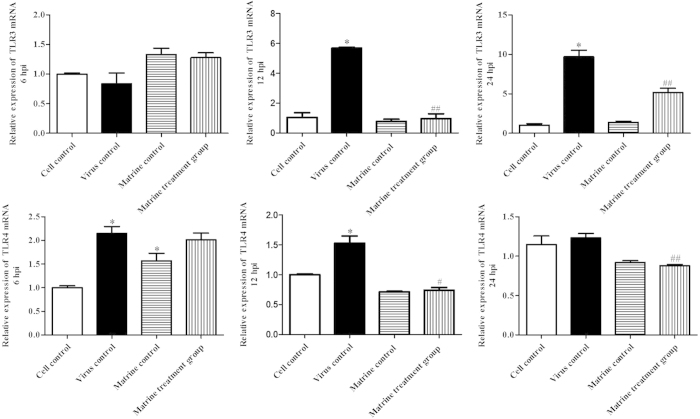
Effect of Matrine on TLR3 and TLR4 expression. PAM cells from all treatment groups were collected and extracted for RNA at 6, 12 and 24 hpi. The mRNA expression of TLR3 and TLR4 was detected using relative quantitative real time PCR. Data were expressed as mean ± standard errors mean (SEM). *indicates that the groups were significant different from cell control (*p* < 0.05). ^#^means the compare between Matrine treatment group and virus control was significant different (*p* < 0.05), ^##^means *p* < 0.01, ^###^means *p* < 0.001.

At 6, 12 and 24 hpi, TLR3 and TLR4 gene in PAM cells from all treatment groups were measured using relative quantitative real time PCR analysis. As shown in Fig. 8, the expression of TLR3 in virus control was much higher than that in cell control at 12 and 24 hpi, indicating that PRRSV/PCV2 co-infection enhanced TLR3 expression. Meanwhile, compared with virus control, the expression of TLR3 was decreased in 0.4 mg/ml of Matrine treatment group. At 6 hpi and 12 hpi, the expression of TLR4 in virus control was much higher than that in cell control, and the Matrine treatment group reduced TLR4 expression at 12 hpi. The expressions of TLR3 and TLR4 were no significant difference between cell control and Matrine alone control at 12 and 24 hpi. All these results indicated that Matrine inhibited virus induced TLR3 and TLR4 expressions.

### Matrine treatment partly block virus induced NF-κB activation

**Figure 9:**
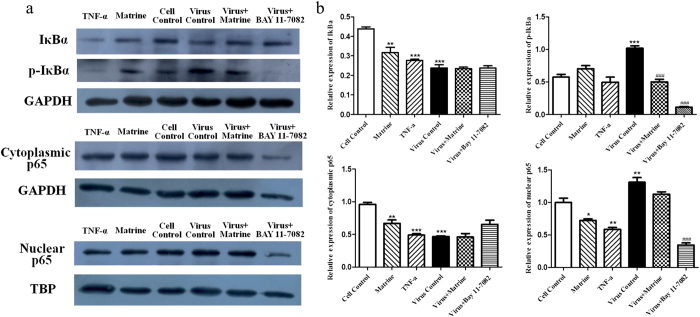
PRRSV and PCV2 co-infection induced NF-κB activation while Matrine treatment barely block virus-induced NF-κB activation. The expressions of IκBα, p-IκBα, cytoplasmic p65 and nuclear p65 were measured by western blot assay (**a**). Quantification of IκBα, p-IκBα, cytoplasmic p65 expression was normalized to GAPDH and the expression of nuclear p65 was normalized to TBP using Image Pro Plus software (**b**). *stands for the groups were significant different with cell control (*p* < 0.05), **means *p* < 0.01, ***means *p* < 0.001. ^#^means the groups were significant different with virus control (*p* < 0.05), ^##^means *p* < 0.01, ^###^means *p* < 0.001.

The degradation and phosphorylation of IκBα and nuclear translocation of p65 are important events in NF-κB signaling activation. According to the results of antiviral assay, we investigated the effect of Matrine on NF-κB pathway at 12 hpi. As shown in Fig. 9, comparing with cell control, the protein levels of IκBα and cytoplasmic p65 in virus group at 12 hpi were significantly decreased, and the levels of p-IκBα and p65 in the nucleus were increased, indicating that PRRSV/PCV2 co-infection induced the translocation of NF-κB from the cytoplasm to the nucleus, indicating the activation of NF-κB pathway. To determine whether Matrine can suppress virus induced NF-κB activation, the protein levels of IκBα, p-IκBα and p65 were examined in Matrine treatment in virus infected PAM cells. The results showed that the expression of IκBα and p65 (both in cytoplasm and nucleus) between virus group and Matrine treatment group were not significantly different except that the p-IκBα expression in Matrine treatment group was remarkably lower than virus group, demonstrating Matrine partly blocked virus induced NF-κB activation. Whilst, BAY11-7082 treatment group significantly decreased IκBα phosphorylation and the p65 expression in the nucleus, indicating 10 μM BAY11-7082 could suppress virus induced NF-κB activation.

To further investigate whether Matrine itself activated NF-κB pathway in PAM cells, PAM cells treated with Matrine or TNF-α were collected. Western blot analysis showed that the protein levels of IκBα and p65 in cytoplasm were decreased both in Matrine group and TNF-α group. These results demonstrated that Matrine at 0.4 mg/ml could trigger IκBα degradation and the nuclear translocation of p65 to induce NF-κB activation.

### Matrine treatment suppressed virus induced TNF-α expression

**Figure 10:**
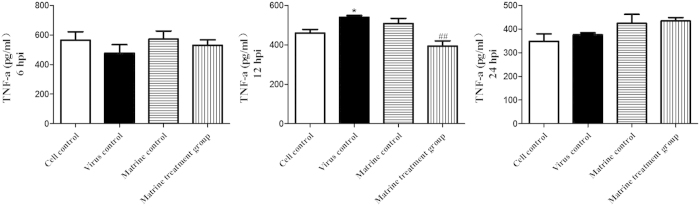
Suppression of virus-induced TNF-α expression by Matrine. ELISA assay was used to measure the expression level of TNF-α at 6, 12 and 24 hpi. Data were expressed as mean ± standard errors mean (SEM). *stands for the groups were significant different with cell control (*p* < 0.05). ^##^means the Matrine treatment group was significant different with virus control (*p* < 0.01).

ELISA assay was used to measure the expression level of TNF-α at 6, 12 and 24 hpi. As shown in Fig. 10, the level of TNF-α in virus control was much higher than that in cell control at 12 hpi (p < 0.05). Whereas, 0.4 mg/ml Matrine treatment group significantly decreased TNF-α expression. Moreover, Matrine itself has no effect on TNF-α expression.

## Discussion

The co-infection of PRRSV and PCV2 is frequently encountered in pig farms worldwide and the PAM cells are the major targets of both PRRSV and PCV2. Effective treatment of the co-infections of these two viruses are still lacking. In present study, we establish a co-infection model for these two viruses *in vitro* by inoculate PAM cells with 10^6^TCID_50_ PRRSV first followed with 10^4^TCID_50_ PCV2. Our qPCR results demonstrated that the infection order used in this study could enhance both PRRSV and PCV2 replication. And the proliferation regularities of PRRSV and PCV2 were essentially consistent in virus alone infected group and PRRSV/PCV2 co-infected group at 6–96 hpi. Our results are resonated by Tsai *et al*.^18^ who mimicked different infection order of dual infections with PRRSV and PCV2. Their results showed that PAM cells inoculated with PRRSV first then inoculated with PCV2 later revealed severe cytopathic effect, and enhanced PRRSV replication which were consistent with the results of Feng *et al*.^19^

We have also investigated the activity of these two viruses in the established PAM model. The PRRSV N gene copies reached the peak at 12 hpi while the PCV2 CAP gene copies were the highest at 72 hpi. After 12 hpi, the PAM cells infected with PRRSV displayed various degree of cell disruption, so the virus was partly released into supernatants which may be as a possible explanation for the PRRSV N gene copies decreased after 12 hpi. We also uncovered that the PCV2 CAP gene copies sharply increased from 6 hpi to 72 hpi and the CAP protein was detected in nucleus. These results provided novel data that PCV2 could complete its replication in PAM cells *in vitro*. Previous work indicated that macrophages are fully differentiated cells which could not provide suitable conditions for completing virus replicative cycle^18^,^19^,^20^. Notwithstanding, Meerts *et al*.^21^ compared the replication kinetics of PCV2 in PAM and PK-15 cells, and showed that the nuclear expression of viral proteins was observed in PAM cells originating from some specific piglets only, demonstrating that the replication of PCV2 dependent on the susceptibility of pigs. Moreover, Williams *et al*.^22^ demonstrated that proliferating cell nuclear antigen (PCNA) positive cells could facilitate the infection of simian immunodeficiency virus to macrophages. PCNA was expressed by proliferating cells in the S phase of cell cycle and non-dividing cells undergoing DNA repair. Macrophages as the terminally differentiated cells have high rates of DNA metabolism. In brief, the mechanisms of PCV2 proliferation in PAM cells are obscured and need to be elaborated in following studies.

The cytotoxicity of compounds should be evaluated prior to investigating their antiviral activities. We have determined that 0.4 mg/ml or less of Matrine are safe for PAM cells. Thus this concentration has been used for investigating antiviral activity of Matrine. Furthermore, we found that the morphological changes induced by Matrine are different from that by Ribavirin. For example, the main cytopathy induced by Matrine was vacuolization which is lacking in Ribavirin. In our previous studies, the MNTC of Matrine to Marc-145 cells^16^ and PK-15 cells^17^ were 0.75 mg/ml and 1 mg/ml, respectively, indicating that the cytotoxicity of Matrine to varied type of cells was different.

Ribavirin is already known to possess broad-spectrum antiviral activity both on RNA and DNA viruses^23^. In this study, we use Ribavirin as a positive control to assess the antiviral activity of Matrine. According to the replication kinetics of PRRSV and PCV2 in PAM cells, the anti-PRRSV/PCV2 co-infection activities of Matrine were assessed at 12 hpi. The results demonstrated that Matrine inhibited both PRRSV and PCV2 infection at 12 hpi. Moreover, the inhibition of PRRSV N gene expression in 0.4 mg/ml Matrine treatment group was much stronger than that in 4 mg/ml Ribavirin treatment group, indicating the antiviral activity of Matrine was better than Ribavirin. Our previous studies showed that Matrine inhibits PRRSV replication in other cell types including Marc-145 cells^16^ and suppress PCV2 replication in PK-15 cells^17^. It can be concluded that Matrine is a promising antiviral compound and further research can be planed to determine the exact mechanism of its antiviral effect of this potent antiviral compound.

Severe interstitial pneumonia was the typical pathology in pigs dually infected with PRRSV and PCV2^24^,^25^. NF-κB signaling pathway plays a vital role in both innate and adaptive immune response as well as inflammatory response to viral infection. Previous researches have been demonstrated that PRRSV or PCV2 infection activated NF-κB pathway both *in vitro* and *in vivo* which in turn lead to virus replication^26^,^27^,^28^. In this study, we examined whether PRRSV/PCV2 co-infection activated NF-κB pathway and the effect of Matrine on NF-κB pathway. BAY 11-7082, a common inhibitor of NF-κB^29^, was used in this study as a control to inhibit NF-κB activation. Our results demonstrated that PRRSV/PCV2 co-infection induced IκBα degradation and phosphorylation as well as the translocation of NF-κB from the cytoplasm to the nucleus, which were consistent with the published results. As Matrine suppressed p-IκBα expression only to a certain degree, it is suggested that Matrine did not completely block virus induced NF-κB activation. The role of NF-κB in antiviral mechanisms of other compounds are demonstrated in two studies. Chen *et al*. reported that *houttuynia cordata* blocked HSV-2 infection through inhibition of NF-κB activation instead of Erk activation^30^. Zhao *et al*. demonstrated that tylvalosin possessed anti-inflammatory activity and attenuated acute lung injury through inhibition of NF-κB activation^31^.

TLRs are one of the upstream regulatory factors of NF-κB pathway. PCV2 infection increased TLR2, TLR3, TLR4 and TLR9 expression in piglet splenic lymphocytes^32^. Infection by PRRSV increased mRNA for TLR3, TLR4 and TLR7 both in the tracheobronchial lymph nodes and brain areas^33^. In this study, we confirmed that PRRSV/PCV2 co-infection increased both TLR3 and TLR4 expression. And we showed that Matrine treatment has decreased virus induced TLR3 and TLR4 expression. In addition, we measured TNF-α expression, a downstream regulatory factors of NF-κB pathway which induces strong proinflammatory response, and the expression of TNF-α could in turn activate NF-κB pathway via a feedback mechanism. We demonstrated that Matrine inhibited virus induced TNF-α expression, indicating the potential of the Matrine in its clinical application.

## Conclusions

Our results revealed that Matrine exhibited antiviral activity against PRRSV/PCV2 co-infection in PAM cells *in vitro*. The underlying antiviral mechanisms of Matrine may be mediated by partly regulating TLR3,4/NF-κB/TNF-α pathway. These results, suggest Matrine and its derivatives have the potential to be developed a new series of drugs against co-infection for clinical application in pig industry.

## Materials and Methods

### Isolation of porcine alveolar macrophages

This experiment was approved by an ethical committee at the Animal Science and Veterinary Medicine College of Shanxi Agriculture University and conducted in compliance with the International Guiding Principles for Biomedical Research Involving Animals (CIOMS and ICLAS, December 2012). Porcine alveolar macrophages (PAMs) were collected from bronchoalveolar lavage fluid according to the description of Sinha *et al*.^34^ with modifications^34^. Briefly, healthy 6-week-old pigs tested PCR-negative for PRRSV and PCV2 and negative for anti-PRRSV and PCV2 antibodies by ELISA were humanely euthanized by intravenous over-dosed pentobarbital sodium. Lungs with trachea were aseptically removed from the thoracic cavity and repeatedly washed by phosphate buffered saline (PBS) through the trachea. The harvested wash fluid containing the PAMs were filtered through 200 mesh nylon net and then centrifuged at 1000 rpm for 5 min. The supernatant was removed and PAM was washed by PBS. Cultures of PAMs with cell viability of more than 95% as assessed by Trypan blue exclusion test were used and the PAMs were adjusted to the density of 2 × 10^7^ cells/ml with pre-cooled medium containing with 70% RPMI 1640, 20% fetal calf serum (FCS), and 10% dimethylsulfoxide (DMSO) and then stored in liquid nitrogen until used.

### Virus titration

The PRRSV (JS-1) used in this study was kindly provided by Prof. G.Q. Shao^35^, and progagated on Marc-145 cells. The stocks of PRRSV with a titer of 10^7.5^TCID_50_/ml were determined by Immunofluorescence assay (IFA) on PAMs. The strain of PCV2 was isolated from a suspected PMWS case at the Veterinary Hospital of Shanxi Agricultural University^17^ and progagated on PK-15 cells. The titer of 10^5.15^TCID_50_/ml for the PCV2 stocks were titrated by IFA on PAMs. The virus titer used in this experiment, was 10^6^TCID_50_/ml for PRRSV and 10^4^TCID_50_/ml for PCV2.

### Determination of Cytotoxicity of Matrine

Matrine and Ribavirin used in this study were purchased from Nanjing Zelang Meditech Ltd. The content of Matrine was 98% by HPLC. The frozen PAMs were quickly thawed at 37 °C water bath and cultured in RPMI-1640 medium supplemented with 10% heat-inactivated FCS. 1 × 10^5^ cells were seeded onto each well of 96-well plate. After 2 h incubation, the non-adherent cells were discarded and the adherent cells were incubated for 24 h in 96-well plates. Matrine and Ribavirin were serially twofold diluted with RPMI-1640 containing 2% FCS and added to the wells. After 48 h incubation, the medium containing the compounds was discarded and 20 μl MTT were then added to each well and incubated for 4 h at 37 °C. The supernatant was then removed and 150 μl DMSO was added to dissolve the formazen crystals. The absorbance was read with an ELISA microplate reader at 490 nm wavelength. During the course of this assay, the effect of compounds on PAM cells morphology was also monitored under light microscope to record any cellular morphological changes. The 50% cytotoxic concentration (CC_50_) value defined as the concentration of compounds that reduced the absorbance of treated cells by 50% relative to cell control were calculated. The maximum non-cytotoxic concentration (MNTC) was calculated as the drug concentration required to maintaining 90% cell viability.

### Establishment of PAM co-infected with PRRSV/PCV2 model

PAM cells were prepared as the described in the section of Determination of Cytotoxicity of Matrine. Four PAM groups were included in this assay: PAM cells control (PAM cells inoculated with an equal volume of maintenance medium containing 2% FCS), PRRSV alone infected group (PRRSV group, PAM cells inoculated with 10^6^TCID_50_ PRRSV 2 h), PCV2 alone infected group (PCV2 group, PAM cells inoculated with 10^4^TCID_50_ PCV2 2 h), and PRRSV and PCV2 co-infected group (PAM cells of PRRSV/PCV2 group were inoculated with 10^6^TCID_50_ PRRSV first then inoculated with 10^4^TCID_50_ PCV2 2 h later). At 6, 12, 24, 48, 72 and 96 h post inoculation (hpi) with PRRSV, PAM cells from all the treatment groups were collected and used for the extraction of RNA and DNA. PRRSV N gene and/or PCV2 CAP gene copy number was determined by absolute quantitative real time PCR assay.

### Antiviral activities of Matrine on PRRSV/PCV2 co-infection

PAM cells were first infected with 10^6^TCID_50_ PRRSV for 2 h, then infected with 10^4^TCID_50_ PCV2 for another 2 h. The viral suspension was removed and different concentrations of Matrine or Ribavirin diluted in fresh medium containing 2% FCS were added. After 12 h incubation, PAM cells from all treatment groups were collected and used for the extraction of RNA to determine the anti-PRRSV activity of Matrine. Moreover, cells were collected to extract DNA at 12 and 72 hpi to evaluate the anti-PCV2 activity of Matrine.

### RNA and DNA extraction and absolute quantitative real time PCR analysis

Nucleic acid extractions from the samples of all treatments (DNA/RNA extraction kit, TianGen, Beijing, China) and cDNA synthesis (Prime Script^TM^ RT reagent Kit with gDNA Eraser) were performed according to the manufacturer’s protocol. The concentrations of nucleic acid were determined using the NanoDrop 1000 spectophotometer (NanoDrop Technologies, Wilmington, DE, USA). Primer sequences and PCR product sizes were described in Table 2. The real time PCR was performed using Applied Biosystems^®^ 7500 Real-Time PCR system, and the PCR was carried out according to the instruction provided by SYBR^®^ Premix Ex Taq™ Kit from TaKaRa. For each experiment, a standard curve was generated using serially diluted plasmid containing N gene or CAP gene.

### Protein extraction and Western blot analysis

After removing the medium, cells were washed twice with PBS. Total cell extracts were prepared with cell lysis buffer (Beyotime Biotechnology, Jiangsu, China) supplemented with protease inhibitors. The cell lysates were centrifuged at 12,000 g for 5 min at 4 °C. The supernatants were collected and stored at −80 °C and protein concentrations were determined using BCA assay. Twenty five (25) μg protein from each sample was separated on 15% sodium dodecyl sulfate polyacrylamide gel electrophpresis (SDS-PAGE) and transferred onto a polyvinylidene fluoride (PVDF) membrane. The membrane was blocked with 5% nonfat milk for 2 h to prevent nonspecific binding and then incubated with anti-PRRSV N monoclonal antibody (1:1000; SDOW17, Rural Technologies) or anti-β-actin antibody (1:4000; CWbio, China) for 2 h at 37 °C. After washed three times with TBST, the membrane was incubated for 1 h with the HRP-conjugated secondary antibody at 37 °C. Following another three time washes with TBST, the protein bands were detected with eECL Western Blot Kit (CWbio Inc., Beijing, China).

### Determination of TLR3 and TLR4 expressions by relative quantitative real time PCR

PAM cells were incubated with 10^6^TCID_50_ PRRSV for 2 h and then incubated with 10^4^TCID_50_ PCV2 for 2 h, and to these cells the maximum non-cytotoxic concentration (MNTC) of Matrine was added. At 6, 12 and 24 hpi, the cells were collected to extract RNA for the real time PCR. Levels of TLR3 and TLR4 mRNA expression were calculated using the 2^−△△CT^ method. Expression of GAPDH was used for normalization.

### TNF-α detection in PAM supernatant

The TNF-α in the supernatant was measured by ELISA according to the manual from the manufacturer (BlueGene Biotech, Shanghai, China). Briefly, cell culture supernatant was centrifuged at 1000 g for 15 min to remove debris. Then 100 μl of TNF-α standards, PBS (control) and samples in triplicate were added to the wells which were pre-coated specific antibody against TNF-α. Ten (10) μl of balance solution was dispensed into each well and mixed. Fifty (50) μl of conjugate was added to the well except for blank control well and then the plates were incubated for 1 h at 37 °C. After incubation, the plates were washed five times with wash solution and then 50 μl substrate A and 50 μl substrate B were added to each well. After further 10 min incubation at 37 °C, 50 μl of stop solution was added to each well to stop the color development. The optical density (OD) was determined at 450 nm using a microplate reader. The standard curve was constructed by plotting the average OD value of each standard on the X axis against the respective concentration on the Y axis. A best fit curve was drawn using ELISA Calc software to generate a four parameter logistic curve-fit. The concentration of TNF-α in a given sample was calculated from the standard curve.

### Detection of IκBα, p-IκBα and p65 by Western blot

PAM cells were inoculated with 10^6^TCID_50_ PRRSV first for 2 h then inoculated with 10^4^TCID_50_ PCV2 for another 2 h, and then the viral inoculum was removed. 0.4 mg/ml Matrine or a specific NF-κB inhibitor (BAY 11-7082, 10 μM) were added to these PAM cells and incubated for 12 h. PAM cells stimulated with 100 ng/ml TNF-α for 30 min were as a positive control. Nuclear and cytoplasmic fractions were isolated using NE-PER^TM^ nuclear and cytoplasmic extraction reagents. Levels of IκBα (1:600; Proteintech), p-IκBα (1:500; Cell Signaling Technology) and p65 (1:1000; Cell Signaling Technology) were measured by Western blot analysis.

### Statistical analysis

The calculation of CC_50_ was performed with nonlinear regression using the function of “log (inhibitor) vs. response-variable slope” as implemented in GraphPad Prism’s. Data used in real time PCR and Western blot were analyzed by one-way ANOVA or t-test implemented in GraphPad Prism 5 software. Data were expressed as mean ± SEM and p < 0.05 was considered to be significant.

## Additional Information

**How to cite this article**: Sun, N. *et al*. Matrine displayed antiviral activity in porcine alveolar macrophages co-infected by porcine reproductive and respiratory syndrome virus and porcine circovirus type 2. *Sci. Rep.* **6**, 24401; doi: 10.1038/srep24401 (2016).

## Figures and Tables

**Figure 1:**
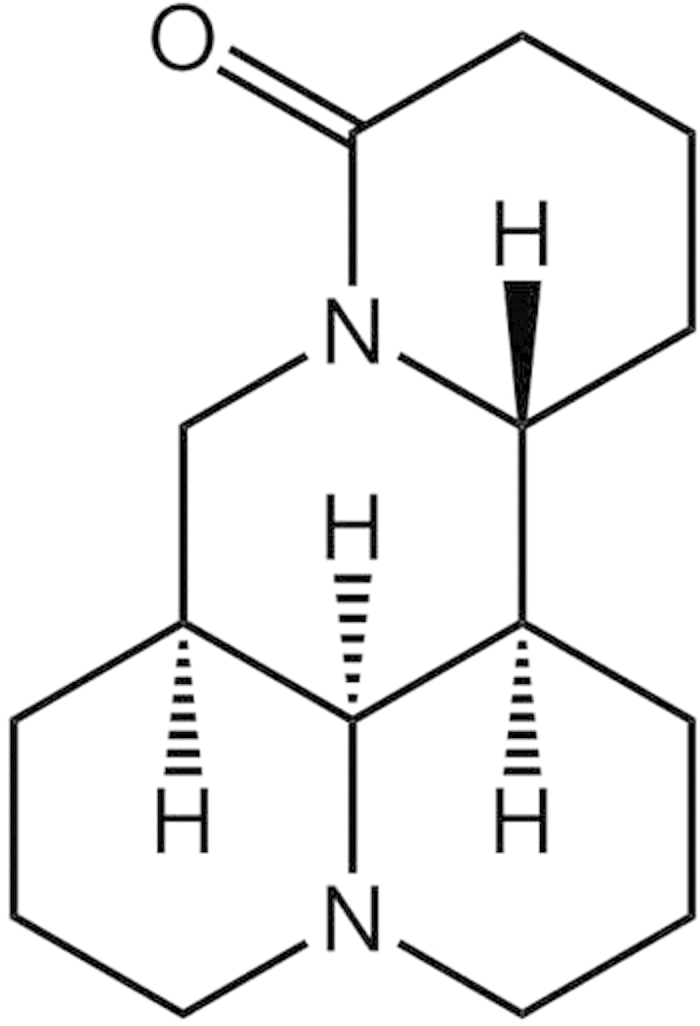
The chemical structure of Matrine.

**Table 1:** The cytotoxicity of Matrine and Ribavirin on PAM cells.

	CC_50_ (mg/ml)	MNTC (mg/ml)
Matrine	0.8681 ± 0.04350	0.4
Ribavirin	5.437 ± 0.2405	4.0

**Table 2:** Primer sequences and the size of PCR products.

Gene	Primer Sequences	PCR products (bp)
PRRSV-N	F: AGAAGCCCCATTTCCCTCTA	196
	R: CGGATCAGACGCACAGTATG	
PCV2-CAP	F: GTCTACATTTCCAGCAGTTTG	148
	R: CTCCCGCCATACCATAA	
TLR3	F: AACTGATGCTCCGAAGGGTG	204
	R: CAGGGTTTGCGTGTTTCCAG	
TLR4	F: TGCTTTCTCCGGGTCACTTC	203
	R: ATGTGGGGATGTTGTCAGGG	
GAPDH	F: TTGGCTACAGCAACAGGGTG	166
	R: CAGGAGATGCTCGGTGTGTT	
